# Genome sequence of OXA-23 producing *Acinetobacter baumannii* IHIT7853, a carbapenem-resistant strain from a cat belonging to international clone IC1

**DOI:** 10.1186/s13099-016-0119-z

**Published:** 2016-07-28

**Authors:** Christa Ewers, Peter Klotz, Sandra Scheufen, Ursula Leidner, Stephan Göttig, Torsten Semmler

**Affiliations:** 1 Institute of Hygiene and Infectious Diseases of Animals, Justus-Liebig-University Giessen, Frankfurter Str. 85-89, 35392 Giessen, Germany; 2 Institute of Medical Microbiology and Infection Control, Hospital of the Johann Wolfgang von Goethe-University, Frankfurt am Main, Germany; 3 Robert-Koch Institute, Berlin, Germany

**Keywords:** OXA-23, *Acinetobacter*, Animal, Carbapenemase

## Abstract

**Background:**

Multidrug resistance in *Acinetobacter baumannii* has dramatically increased in recent years worldwide. Thus, last-line antibiotics like carbapenems are increasingly being used which in turn further augments selection pressure for resistant strains. Resistance to carbapenems in *A. baumannii* is frequently mediated by carbapenemases, particularly OXA-23 and OXA-58. Carbapenemase-producing bacteria are mainly described in human patients and the intestinal tract represents a common source for such pathogens. In this study, we sequenced and analyzed the genome of *A. baumannii* IHIT7853, a carbapenem-resistant, OXA-23 producing strain isolated from cystitis in a cat in 2000 in Germany.

**Results:**

Phylogenetic analysis revealed that IHIT7853 belonged to the globally distributed international clone IC1 and MLST type ST1/ST231 (Pasteur/Oxford MLST scheme). A phylogenetic tree based on the maximum common genome of 18 *A. baumannii* isolates placed IHIT7853 close to human clinical isolates, such as the multidrug-resistant (MDR) outbreak strain AYE that was isolated from a patient with pneumonia and cystitis in 2001 in France. The OXA-23 plasmid sequence could be determined as 53,995 bp in size, possessing resistance genes *strA* and *strB* in addition to *bla*_OXA-23_.

**Conclusions:**

The analysis of the genome of IHIT7853 reveals that companion animals carry MDR *A. baumannii* that resemble relevant clonal lineages involved in severe infections in humans. As urinary tract infections are often caused by bacteria that reside in the intestinal tract, future studies should unveil, if the animal gut serves as a source for MDR *A. baumannii*.

## Background

*Acinetobacter baumannii* is widely recognized as a nosocomial pathogen that is characterized by its intrinsic and acquired antimicrobial resistance. Of particular concern in hospital settings is the development of multidrug-resistant (MDR) and particularly of carbapenem-resistant strains, which have increased markedly in recent years [1]. The intestinal microbiota forms a major reservoir of *A. baumannii* and various infections, including bacteraemia, cystitis and wound infections can occur once the bacteria translocate across the intestinal barrier or reach the patient’s skin as a consequence of faecal shedding [2, 3]. The finding of identical strains in the blood and gastrointestinal tract of patients with *A. baumannii* bloodstream infections strengthens the role of the gut as an important source of the pathogen [2].

Carbapenem resistance in *A. baumannii* is most frequently mediated through intrinsic (OXA-51-like) or acquired (e.g. OXA-23, OXA-58) carbapenem-hydrolyzing class D β-lactamases (CHDLs) that confer resistance once they are overexpressed [4]. OXA-23-producing *A. baumannii* contribute to hospital-outbreaks worldwide and have recently tended to replace OXA-58-producing strains in some countries [1, 5–8]. In Germany, the first outbreak of *A. baumannii* carrying the carbapenemase OXA-23 was observed in 2006 and nowadays it accounts for about 80 % of MDR *A. baumannii* in Germany [7, 9]. Until recently, carbapenem-resistant *A. baumannii* have been mainly described in human patients. However, previous studies have identified this species as an emerging pathogen particularly in hospitalized companion animals and horses [9–11]. Here, we report the genome sequence of the carbapenem-resistant *A. baumannii* strain IHIT7853 that was isolated from the urine of a hospitalized cat with cystitis.

## Methods

### Bacteria, DNA isolation, genetic relatedness and antimicrobial susceptibility testing

*Acinetobacter baumannii* strain IHIT7853 was isolated from the urine of a cat admitted to a veterinary clinic in November 2000. For genomic DNA extraction late log-phase cells were harvested and lysed with EDTA, lysozyme, and detergent treatment, followed by proteinase K and RNase digestion using the DNeasy Blood & Tissue Kit (Qiagen, Germany) according to the manufacturer´s recommendation. Genomic DNA yield, purity, and concentration was evaluated using 0.7 % agarose gel electrophoresis with λ-Hind III DNA marker (Thermo Fisher Scientific, USA). The genomic DNA was stored at −20 °C until use. Genetic relatedness of *A. baumannii* isolates was investigated by semi-automated repetitive element palindromic (rep)-PCR using the DiversiLab^®^ strain typing platform (BioMérieux, Nürtingen, Germany) as previously described [7]. Results were utilized with the DiversiLab^®^ software using Pearson correlation to determine distance matrices and the unweighted pair group method with arithmetic averages to create dendrograms. MIC data were generated using the VITEK 2 system and AST-GN38 testing cards (BioMérieux).

### Genome and plasmid sequencing and annotation

Whole-genome sequencing of *A. baumannii* strain IHIT7853 was performed on an Illumina MiSeq (MiSeq Reagent Kit v3, Illumina Inc.) resulting in 300 bp paired-end reads and an average coverage of 130×. De novo assembly after quality trimming of the reads was conducted using CLC Genomics Workbench v. 9 (CLC bio, Denmark) with standard parameters, scaffolding and exclusion of contigs smaller than 200 bp. The genome was annotated using the Rapid Annotation using Subsystem Technology (RAST) server [12].

Functional classification of the genes was conducted by BLASTP with the Clusters of Orthologous Groups of proteins (COG) database [13]. Prediction of phage sequences and clustered regularly interspaced short palindromic repeats (CRISPRs) was performed with PHAST [14] and CRISPRfinder [15]. Antimicrobial resistance genes and insertion sequences were identified using the CGE website [16] and ISfinder, respectively [17].

Strain IHIT7960 was isolated from a dog in the same clinic where IHIT7853 has been obtained, and proved to be a natural plasmid-free, isogenic variant of *A. baumannii* IHIT7853. IHIT7960 was whole genome sequenced and de novo assembled as mentioned before. Whole genome sequencing reads from the plasmid-containing strain IHIT7853 were mapped against the genome of IHIT7960 and all reads that could not be mapped, and therefore represent plasmid DNA, were de novo assembled as described above. Few reads that could not be mapped during de novo assembly but did not represent plasmid sequences were not used to generate the plasmid contig.

### Phylogenetic analysis

For comparative genomic analysis, genome sequences of representative *A. baumannii* strains were downloaded from the NCBI website: ATCC 17978 (CP012004), LAC-4 (CP007712), ABBL102 (LLHC00000000), TYTH-7 (AGSV00000000), GTC 03328 (BBNI00000000), RUH1486 (JZBU00000000), AB030 (CP009257), MDR-ZJ06 (CP001937), MDR-TJ (CP003500), NCGM 237 (AP013357), NIPH 1669 (APOQ00000000), AB307-0294 (NC_011595), strain A1 (CP010781), AYE (CU459141.1), AB0057 (NC_011586.1), TTU2014-131AME (LKJZ00000000), and FDAARGOS_123 (LORJ00000000). The source of the strains is given in Fig. 3. MLST types were determined following the Institute Pasteur [18] and Oxford [19] approach and using the CGE server [16]. The determination of the maximum common genome (MCG) alignment was done comprising those genes present in all 18 downloaded genomes [20]. For this purpose, we clustered the coding sequences based on the parameters sequence similarity (min. 70 %) and coverage (min. 90 %) and defined the genes that were present in each genome, thereby fulfilling the threshold parameters as MCG. This resulted in 2480 orthologous genes which we used for comparisons. Allelic variants of these genes from all genomes were then extracted by a BLAST-based approach, aligned individually for each gene and concatenated. This resulted in an alignment of 2.157 Mbp for the 18 *A. baumannii* strains. The alignment was used to generate a phylogenetic tree with RAxML 8.1 [21] using a General Time Reversible model and gamma correction for among site rate variation.

### Quality assurance

*Acinetobacter baumannii* IHIT7853 that was obtained from a single colony was maintained at the Institute of Hygiene and Infectious Diseases, Giessen, Germany, and genomic DNA was extracted from a pure culture. The 16S rDNA from the draft genome was used to check for contamination. *GyrB* gene sequencing and VITEK 2 biochemical identification (BioMérieux, Nürtingen, Germany) confirmed that the strain IHIT7853 belonged to the species *A. baumannii*. Possible contamination with other genomes and misassemblies were checked by mapping the reads back to the contigs. The read mapping of the draft genome of IHIT7853 is in the range of expected size distribution and the coverage of the reads was consistent throughout the genome.

## Results and discussion

### General features

Filtered 2.56 M clean reads were assembled into scaffolds, and corresponding 130-fold coverage of the genome was generated. The draft genome sequence of *A. baumannii* IHIT7853 was 4,221,000 bp in size and had a G + C content of 39.29 % in 372 contigs, with N50 spanning 223,827 bp (Table 1). Annotation of this assembly identified 3960 coding sequences (CDSs), 65 tRNAs, and 12 rRNAs.

**Table 1:** General features of the *A. baumannii* IHIT7853 genome

Item	Value
Number of contigs	372
Total contig length (bp)	4,220,991
Fold coverage (x)	130
N50 (bp)	223,827
G + C content (%)	39.29
Number of protein coding genes	3960
Number of predicted transfer RNAs	65
Number of predicted ribosomal RNAs	12
GenBank accession number	LWTH00000000

**Fig. 1:**
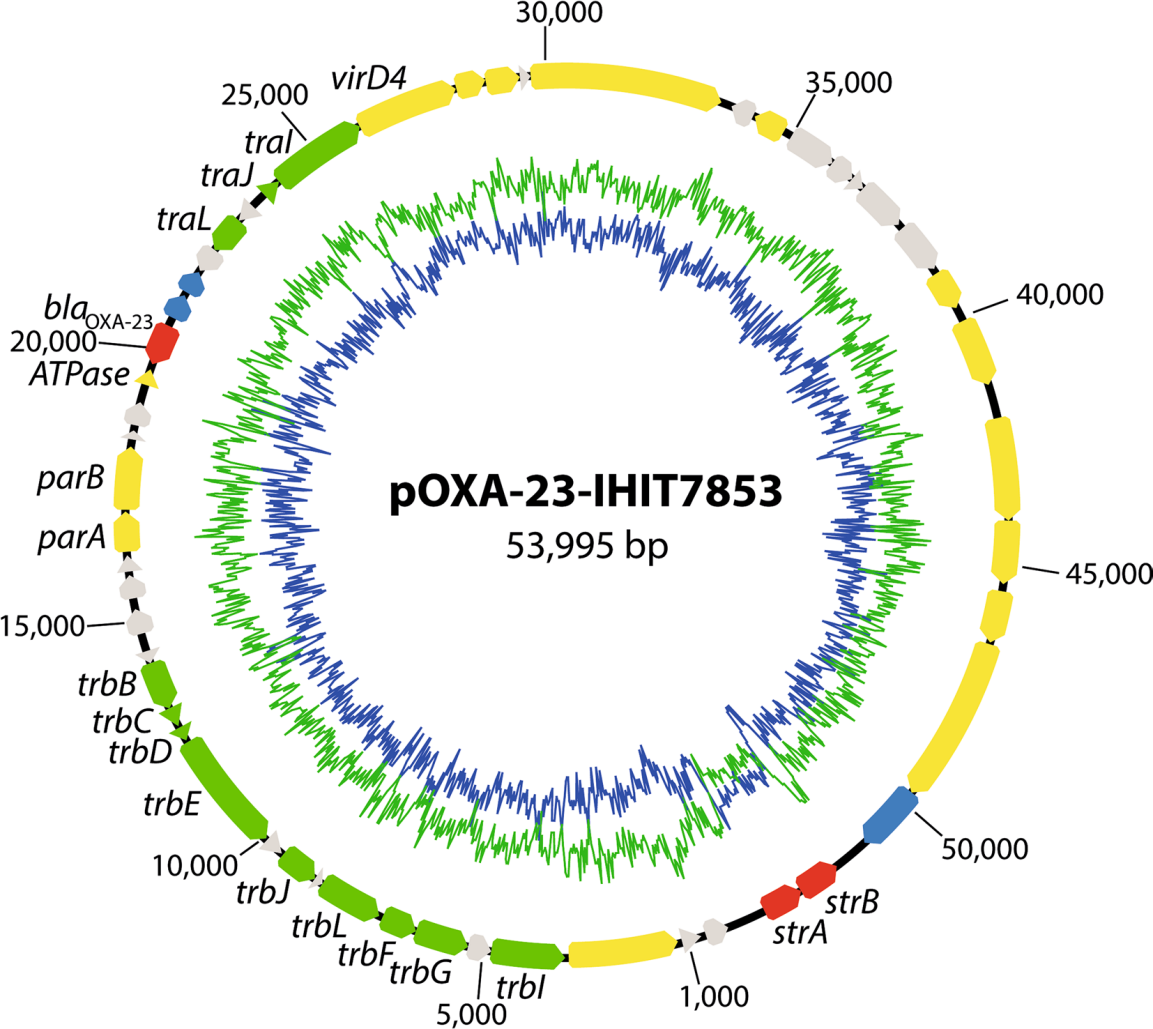
Circular map of plasmid pOXA-23-IHIT7853 of *A. baumannii* strain IHIT7853 generated with Geneious 8.1.3 (Biomatters Ltd, Auckland, New Zealand). *Broad arrows* in the outer circle indicate genes coding for resistance factors (*red*), transfer proteins (*green*), hypothetical proteins (*grey*), insertion sequences/mobile elements (*blue*), and other factors (*yellow*), as partially indicated in the figure. *Inner circles* represent G + C content (*blue*) and AT graph (*green*)

The reads belonging to the plasmid could be assembled into one single contig of 53,995 bp in size (Table 2; Fig. 1). The plasmid sequence had a G + C content of 36.5 % and revealed 53 protein coding sequences.

**Table 2:** General features of the pOXA-23-IHIT7853 plasmid

Item	Value
Number of contigs	1
Total contig length (bp)	53,995
Fold coverage (x)	450
G + C content (%)	36.5
Number of protein coding genes	53
GenBank accession number	KX118105

**Fig. 2:**
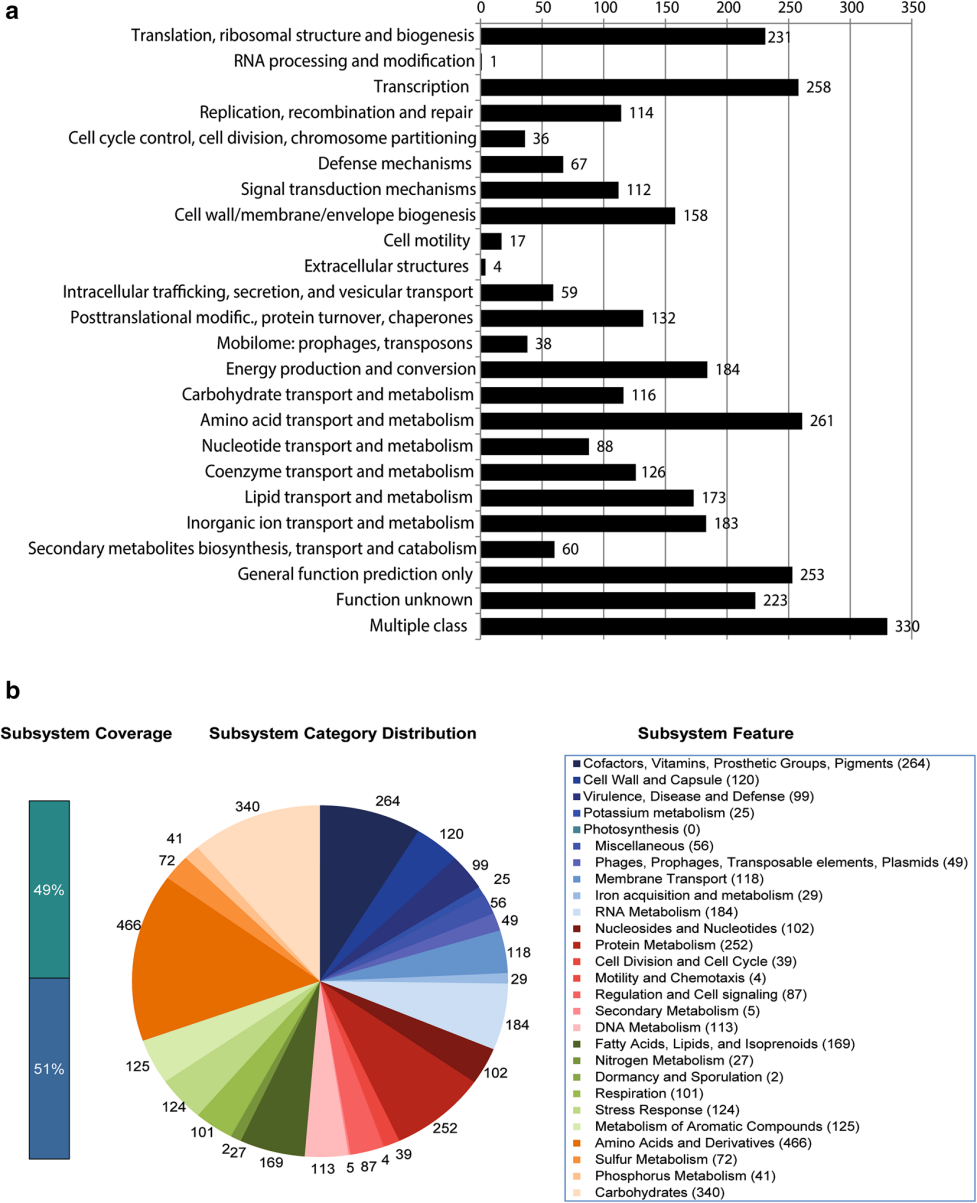
COG analysis and subsystem distribution. **a** COGs distribution of *A. baumannii* strain IHIT7853; **b** distribution of genes assigned to SEED subsystems (based on the RAST annotation server)

We categorized 3224 genes into COGs functional groups, including putative or hypothetical genes and genes of unknown function. For COGs distribution, abundant categories were amino acid transport and metabolism (261 ORFs), transcription factors (258 ORFs), general function prediction only (253 ORFs), and translation, ribosomal structure and biogenesis (231 ORFs) (Fig. 2a). The subsystem distribution and general information on the potential functional distribution of *A. baumannii* IHIT7853 is illustrated in Fig. 2b. Genes associated with amino acids and derivatives (466 ORFs), carbohydrates (340 ORFs), cofactors, vitamins, prosthetic groups, and pigments (264 ORFs), and protein metabolism (252 ORFs) were abundant among the SEED subsystem categories.

Five prophage regions have been identified in the genome of IHIT7853, of which 2 regions are intact and 3 regions are incomplete. Intact phages are (i) phi CTX, a P2-like cytotoxin-converting phage of *Pseudomonas aeruginosa* (Acc-No. NC_003278) of 34.1 kb in length and a G + C content of 38.72 % and (ii) *Acinetobacter* phage YMC/09/02/B1251_ABA_BP (NC_019541), which is 45.36 kb in length, reveals a G + C content of 39.05 % and has previously been demonstrated to cause lysis of an OXA-23-producing carbapenem-resistant *A. baumannii* isolate from a septic patient [22]. One CRISPR candidate was detected consisting of 28 bp-repeat-areas and 54 spacers. Located downstream of the repeat and spacer region are the *cas* genes *csy4—csy1*, *cas3*, and *cas1*. Several insertion sequence (IS) elements, including IS*Aba1*, IS*Aba12*, IS*15*, IS*17*, and IS*26* were identified using IS Finder.

The *bla*_OXA-23_ gene of *A. baumannii* IHIT7853 was localized on a 53.99 kb plasmid that also encoded aminoglycoside resistance genes *strA* and *strB*. Other resistance genes detected in the genome of IHIT7853 were aminoglycoside resistance genes *aadA1*, *aph(3´)* and *aac(3)-Ia*, β-lactamase gene *bla*_OXA-69_ (lacking an upstream insertion sequence), phenicol resistance gene *catA1*, and the genes *sul1* and *tet(A)* coding for sulfonamide and tetracycline resistance, respectively. In agreement with these genetic features, high MICs were identified for imipenem (≥16 mg/L), piperacillin (≥16 mg/L), cefpirome (≥64 mg/L), gentamicin (≥16 mg/L), tetracycline (≥16 mg/L), and trimethoprim/sulfamethoxazole (≥32 mg/L). MICs to enrofloxacin (2 mg/L) and marbofloxacin (≥4 mg/L) are in correspondence with the finding of a single mutation in the *gyrA* sequence, resulting in an amino acid change from serine to leucine at position 83.

### Phylogenetic analysis

**Fig. 3:**
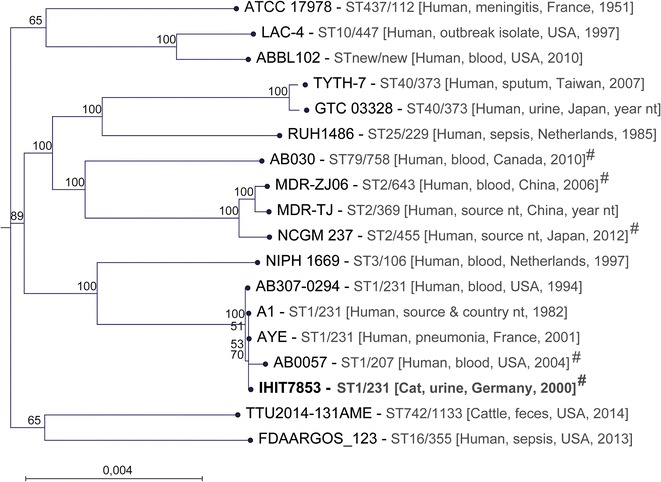
Maximum likelihood tree (based on 2480 orthologous genes) of 18 *A. baumannii* strains. The *scale* indicates substitutions per site. Bootstrap values are based on 100 iterations. # indicates the presence of a *bla*_OXA-23_ gene in the strains. The text in brackets provides information about host, sample material or clinical source, country, and year of isolation

*Acinetobacter baumannii* strain IHIT7853 was determined as sequence type ST1^P^ and ST231^Ox^ according to the Pasteur and Oxford MLST scheme, respectively. Genetic relatedness of IHIT7853 to other carbapenem-resistant *A. baumannii* clinical isolates which were collected during a 12-year period was analyzed by semi-automated rep-PCR. IHIT7853 showed highest similarities to two Oxa-23 positive isolates from human patients which were isolated from rectal swab and an intraoperative gut wound swab, respectively (data not shown). Clustering analysis showed that IHIT7853 belongs to the international clone IC1 in accordance with the fact that ST1 strains are member of this lineage. Together with IC2, this clonal lineage accounts for most of the nosocomial and community-acquired infections with *A. baumannii* worldwide [7]. Based on genomes from published *A. baumannii* strains from humans and on one genome available from cattle, we performed phylogenetic analysis on tree topology to identify genetic relatedness of the strains. IHIT7853 is highly similar to strain AYE, a MDR strain cultured from a patient with pneumonia and a urinary tract infection during an outbreak of ESBL- and VEB-1-producing *A. baumannii* isolates in a French hospital in 2001 (Fig. 3) [23]. Other human ST1/ST231 strains are also highly related to the animal strain suggesting that a transmission of such MDR strains may likely occur between animals and humans in both directions.

## Conclusions

This is the first description of the genome and the OXA-23 plasmid sequence of a carbapenem-resistant *A. baumannii* from an animal. So far, whole genome studies of *A. baumannii* have nearly exclusively focused on comparing strains from human patients. Genome data of IHIT7853 revealed high relatedness of the cat strain to human clinical strains providing evidence for a possible transmission of these bacteria between humans and animals. Based on knowledge from human patients it should be considered that the gastrointestinal tract of animals might serve as a source of MDR *A. baumannii* probably leading to infections at other sites [2, 3]. Future studies should unveil epidemiologic links between human and animal strains and track the genome dynamics in MDR *A. baumannii* from different hosts as well as from colonization and infection sites.

## Abbreviations

COG: clusters of orthologous groups of proteins
CRISPR: clustered regularly interspaced short palindromic repeats
IC: international clone
IS: insertion sequence
ST: sequence type
